# Targeted hip abductor fatigue alters trunk and lower limb biomechanics during Single-Leg landing

**DOI:** 10.1038/s41598-025-20279-0

**Published:** 2025-10-16

**Authors:** Kengo Harato, Kohei Nishizawa, Satoshi Imai, Shu Kobayashi, Kazuya Kaneda, Tatsuaki Matsumoto, Takeo Nagura

**Affiliations:** 1 Sports Medicine Research Center, Keio University, Yokohama City, Kanagawa prefecture, Japan; 2 Department of Orthopaedic Surgery, Keio University School of Medicine, Tokyo, Japan; 3 Institute for Integrated Sports Medicine, Keio University School of Medicine, Tokyo, Japan; 4 Department of Clinical Biomechanics, Keio University School of Medicine, Tokyo, Japan; 5 Sports Medicine Research Center, Keio University, Hiyoshi 4-1-1, Kohokuku, Yokohama City, Kanagawa prefecture, 223-8521 Japan

**Keywords:** Knee ligament injury, Trunk control, Neuromuscular adaptation, Joint loading, Landing strategy, Risk factors, Motor control

## Abstract

Fatigue of the hip abductor muscles may influence lower limb biomechanics and potentially contribute to anterior cruciate ligament (ACL) injury risk. However, the effects of targeted hip abductor fatigue on trunk, pelvis and lower limb coordination during landing tasks remain unclear. The present study aimed to investigate how targeted hip abductor fatigue alters the biomechanics of the trunk, pelvis, and lower extremity during single-leg landing (SLL). Twenty healthy male recreational athletes performed SLL before and after a targeted hip abductor fatigue protocol. Kinematic and kinetic data were collected using a three-dimensional motion capture system and force plates, with analysis focused on the timing of peak vertical ground reaction force. Following fatigue, participants exhibited increased hip abduction angle and trunk right inclination, as well as decreased hip flexion and external hip abduction moment. Notably, the external knee abduction moment significantly increased post-fatigue, though it remained negative in absolute value. Increased pelvic left rotation was also observed, indicating compensatory adjustments in trunk-pelvis coordination. These adaptations indicate that targeted muscle fatigue induces complex biomechanical responses across the kinetic chain, rather than uniformly increasing injury risk through valgus-prone mechanics. While the observed changes may reflect stabilizing strategies under fatigue, their implications for ACL loading require further clarification. The current study highlights the relevance of considering whole-body biomechanical responses to targeted fatigue and contributes to a more detailed understanding of neuromuscular control during landing. These findings may support the refinement of injury prevention approaches that address segmental coordination under fatigued conditions.

## Introduction

Fatigue has been proposed as a potential risk factor for noncontact anterior cruciate ligament (ACL) injuries due to its influence on neuromuscular control and joint biomechanics^1–5^. Among various contributing factors, weakness or fatigue in the hip abductors has been implicated in altering frontal plane mechanics, potentially increasing knee abduction angle and moment. The hip abductor muscles are critical in stabilizing the pelvis and femur during dynamic tasks, such as single-leg landing (SLL), by resisting excessive contralateral pelvic drop and controlling hip adduction^6,7^. Clinically, approximately 70% of ACL injuries occur during noncontact situations, including deceleration, pivoting, or landing maneuvers^8–10^. Biomechanical studies have identified excessive knee abduction angle and moment as key risk factors associated with these injury mechanisms^11–14^.

Previous studies have primarily focused on lower limb biomechanical consequences, particularly at the knee and hip levels^15–17^. Several studies, including those by Patrek et al., Kim et al., and Geiser et al., have specifically examined the impact of isolated hip abductor fatigue on lower limb biomechanics^15–17^. These studies reported changes such as increased hip abduction and reduced external knee abduction moments, suggesting unexpected adaptations contrary to the conventional assumption that fatigue mimics muscle weakness and thus leads to riskier movement patterns. However, the influence of hip abductor fatigue on trunk and pelvis biomechanics—key components of the kinetic chain—remains poorly understood. Trunk positioning has been shown to influence lower limb mechanics and ACL loading, and poor core control has been associated with increased ACL injury risk^18,19^. Sakurai et al. demonstrated that foot positioning could significantly alter trunk and pelvic kinematics during SLL^20^. Thus, it is necessary to evaluate the biomechanical adaptations across the entire kinetic chain.

The present study aimed to investigate the effects of targeted hip abductor fatigue on the biomechanics of the trunk, pelvis, and lower extremity during SLL. It was hypothesized that targeted hip abductor fatigue would result in altered multi-segmental coordination, potentially contributing to movement patterns associated with increased knee ligament injury risk.

## Methods

Twenty healthy, male recreational-level athletes (mean age: 19.6 ± 1.1 years; weight: 64.3 ± 4.3 kg; height: 1.73 ± 0.04 m) participated in the present study. All participants had a Tegner activity level of 7. Nine participants were members of a university ski club and eleven were members of a soccer club. Each had training session 3–4 times per week for approximately 3 h per session. Only males were included to control for sex-related differences in biomechanics and injury mechanisms. All participants provided informed consent as approved by our institutional review board.

**Fig. 1:**
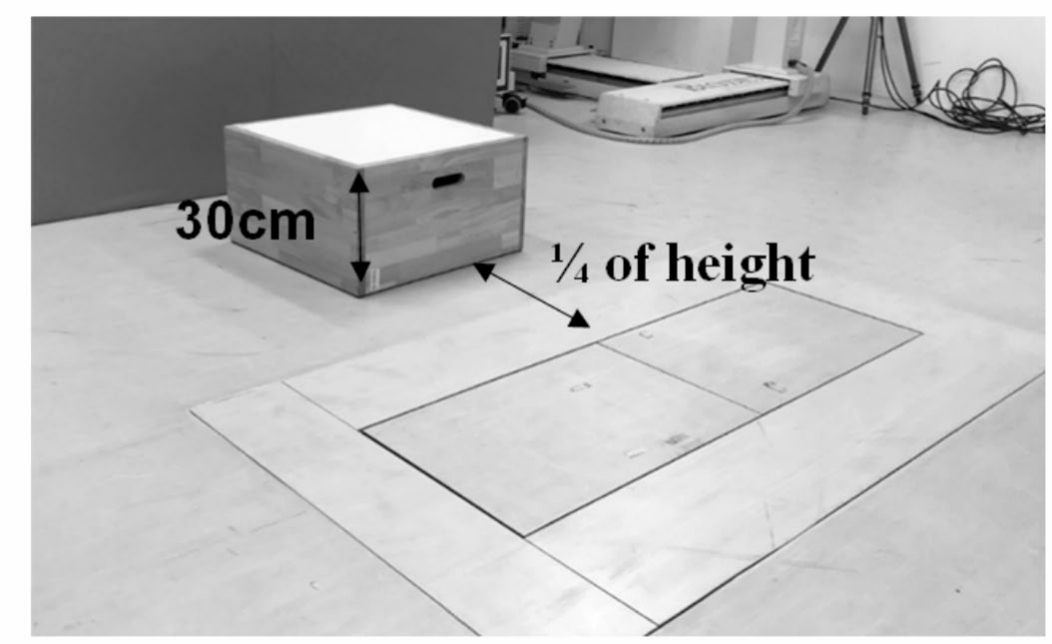
Experimental setup for single-leg landing tasks. Participants jumped from a 30-cm high box to a target 25% of their height away from the box, landing on force plates

Participants performed SLL tasks before and after a fatigue protocol targeting the hip abductor muscles. Before performing the SLL tasks, participants received standardized verbal instructions and visual demonstrations, followed by 2–3 practice trials to ensure task familiarity and to serve as a warm-up. The SLL task involved jumping from a 30-cm box to a target located at a distance equal to 25% of the participant’s height, landing on a force platform (Fig. 1)^20^.

**Fig. 2:**
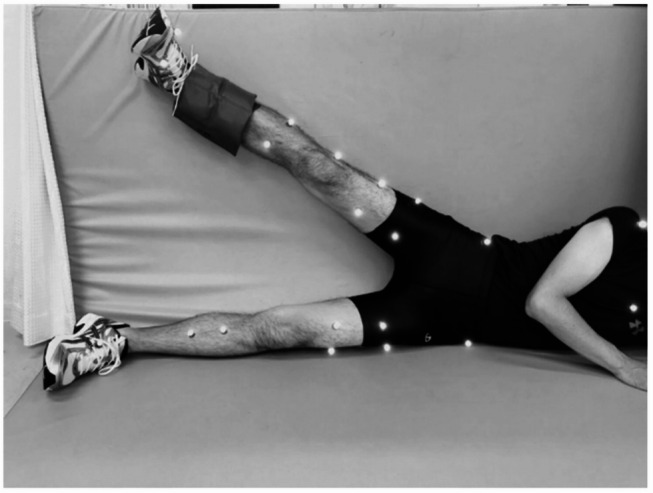
Hip abductor fatigue protocol. Participants performed targeted hip abduction movements with incremental resistance until exhaustion

Fatigue was induced using a targeted hip abductor protocol. Participants lay in a side-lying position and performed hip abduction exercises against a 10-kg weight attached to the distal lower limb until they were unable to complete a full range of motion (defined as failure). This was followed by a second set using 5-kg resistance until additional failure occurred (Fig. 2). Although this protocol primarily targets the gluteus medius, involvement of other synergistic muscles cannot be excluded. The dominant leg (all right) was used for both fatigue induction and landing analysis.

Three-dimensional motion capture was conducted using eight infrared cameras (120 Hz; ProReflex, Qualisys, Sweden) and two synchronized force plates (600 Hz; AM6110, Bertec, USA). A total of 46 retro-reflective markers were placed according to a standardized lower extremity and trunk model described in the previous study^20^ (Fig. 2). Data were processed using Visual 3D (C-Motion, Rockville, MD, USA). Marker trajectories and force plate data were low-pass filtered using a fourth-order Butterworth filter (cutoff: 6 Hz for kinematics, 30 Hz for kinetics).

Joint kinematics (flexion, abduction, internal rotation angles) and kinetics (external joint moments) of the hip and knee were calculated at the timing of peak vertical ground reaction force (vGRF). Trunk and pelvic angles in the sagittal, frontal, and transverse planes were also computed. These angles were calculated using a Cardan XYZ sequence (flexion/extension, lateral flexion, axial rotation), based on each segment’s local coordinate system relative to the global coordinate system, as described in our previous study^20^. The global coordinate system was defined with the X-axis pointing anteriorly, the Y-axis pointing vertically upward, and the Z-axis pointing to the right of the participant. All joint moments were expressed in the external sense, with moments exported from Visual 3D inverted in sign where necessary.

Statistical comparisons between pre- and post-fatigue conditions were performed using paired two-tailed t-tests. Prior to these analyses, the normality of each variable was assessed using the Shapiro–Wilk test. All variables satisfied the assumption of normality except for the external hip external rotation moment, for which a non-parametric two-tailed Wilcoxon signed-rank test was applied. Statistical significance was set at *p* < 0.05. Effect sizes were calculated using Cohen’s d and categorized as small (0.2), medium (0.5), or large (0.8). All statistical analyses were performed using SPSS^®^ for Windows (version 29; Microsoft, Chicago, IL).

## Results

Peak vertical ground reaction force (vGRF), normalized to body weight, was 4.59 ± 0.71 before the fatigue protocol (FP) and 4.47 ± 0.75 after FP, with no significant difference between conditions (*P* = 0.42). The peak anteroposterior ground reaction force was significantly reduced following FP (0.04 ± 0.08) compared to pre-FP (0.10 ± 0.08, *P* = 0.021), whereas the peak mediolateral ground reaction force showed no significant change (0.26 ± 0.17 vs. 0.24 ± 0.18, *P* = 0.58).

Three-dimensional knee joint angles did not differ significantly between conditions (Table 1). In contrast, hip flexion angle significantly decreased post-FP (*P* = 0.014), while hip abduction angle significantly increased (*P* < 0.01). Regarding kinetics, the external knee abduction moment significantly increased after FP (− 0.61 ± 0.44 to − 0.36 ± 0.50 Nm/kg, *P* = 0.037), although values remained negative. The external hip abduction moment significantly decreased post-FP (0.27 ± 1.1 to − 0.53 ± 1.2 Nm/kg, *P* < 0.01).

**Table 1:** Biomechanical parameters of hip/knee joints at the timing of peak vertical GRF during single-leg landing before and after fatigue protocol (FP)

	Pre-FP	Post-FP	*P* Value^a^	Effect size^b^
Knee flexion angle (Deg.)	33.4 ± 4.8	31.5 ± 4.6	0.082	0.41
Knee abduction angle (Deg.)	3.9 ± 4.0	3.8 ± 4.2	0.84	0.045
Tibial internal rotation angle (Deg.)	−0.44 ± 6.3	−0.19 ± 7.0	0.71	−0.083
External knee flexion moment (Nm/kg)	1.7 ± 1.0	1.5 ± 0.9	0.36	0.21
External knee abduction moment (Nm/kg)	−0.61 ± 0.44	−0.36 ± 0.50	0.037	−0.50
External knee internal rotation moment (Nm/kg)	0.093 ± 0.31	0.0050 ± 0.26	0.24	0.27
Hip flexion angle (Deg.)	33.9 ± 9.2	30.8 ± 10.9	0.014	0.60
Hip abduction angle (Deg.)	9.1 ± 5.5	12.1 ± 6.8	< 0.01	−0.72
Hip internal rotation angle (Deg.)	−6.0 ± 8.3	−8.4 ± 9.8	0.089	0.40
External hip flexion moment (Nm/kg)	5.2 ± 2.0	4.9 ± 1.9	0.60	0.12
External hip abduction moment (Nm/kg)	0.27 ± 1.1	−0.53 ± 1.2	< 0.01	0.68
External hip external rotation moment (Nm/kg)	−0.79 ± 0.55	−0.71 ± 0.52	0.46*	−0.16

^a^ Values obtained using Paired t-test except for External hip external rotation moment

^b^ Values of Cohen’s d

* Two-tailed Wilcoxon signed-rank test was used only for External hip external rotation moment.

With respect to trunk and pelvic kinematics (Table 2), trunk right inclination significantly increased following FP (*P* = 0.01). No significant pelvic drop was observed. Additionally, left pelvic rotation was significantly greater post-FP compared to pre-FP (*P* < 0.01).

**Table 2:** Biomechanical parameters of trunk and pelvis at the timing of peak vertical ground reaction force (vGRF) during single-leg landing before and after fatigue protocol (FP).

	Pre-FP	Post-FP	*P* Value^a^	Effect size^b^
Anterior inclination of trunk (Deg.)	7.5 ± 9.2	7.7 ± 8.1	0.89	−0.03
Right inclination of trunk (Deg.)	2.7 ± 3.1	4.2 ± 2.3	0.01	−0.64
Left rotation of trunk (Deg.)	1.6 ± 6.0	2.0 ± 6.1	0.81	−0.06
Anterior inclination of pelvis (Deg.)	8.5 ± 8.2	7.4 ± 8.9	0.20	0.30
Right inclination of pelvis (Deg.)	4.6 ± 3.2	5.5 ± 3.2	0.26	−0.26
Left rotation of pelvis (Deg.)	7.9 ± 6.9	13.3 ± 9.1	< 0.01	−0.72

^a^ Values obtained using two-tailed Paired t-test

^b^ Values of Cohen’s d

## Discussion

The present study investigated the biomechanical adaptations induced by targeted hip abductor fatigue during single-leg landing (SLL), with particular focus on multisegmental coordination involving the trunk, pelvis, and lower extremity. The results confirmed our hypothesis that targeted fatigue would alter segmental strategies. Specifically, post-fatigue landing was characterized by increased hip abduction angle, decreased external hip abduction moment, increased trunk right inclination, and increased pelvic left rotation. These findings indicate that compensatory strategies are employed in response to targeted muscular fatigue, affecting the entire kinetic chain. Collectively, these changes represent an integrated multi-joint response involving the trunk, pelvis, hip, and knee, highlighting a kinetic chain adaptation to targeted muscular fatigue. These findings should not be interpreted as isolated segmental events, but rather as components of a coordinated kinetic chain response. Fatigue at the hip abductor level likely disrupted proximal control, triggering a cascade of compensatory adjustments across the trunk, pelvis, and distal joints. For example, loss of active hip stabilization may have necessitated increased trunk lean and pelvic rotation to maintain balance, which in turn influenced loading patterns at the knee. This proximal-to-distal propagation is consistent with kinetic chain theory and underscores the importance of viewing landing biomechanics as an integrated system.

Importantly, we observed a significant increase in the external knee abduction moment following fatigue, despite values remaining in the negative (valgus-directed) range. This result contrasts with earlier studies by Kim et al., Patrek et al., and Geiser et al., which reported either unchanged or reduced knee abduction moments following similar fatigue protocols^15–17^. One possible explanation for this discrepancy is that our participants—healthy young males—may have adopted different compensatory strategies, such as increased trunk right inclination and pelvic left rotation, which could redistribute load across the lower limb. In contrast, prior studies often included female athletes or mixed populations, and differences in neuromuscular control strategies may have influenced the biomechanical responses. The present finding suggests that, under certain fatigue conditions, increased frontal plane knee loading may occur. While the external knee abduction moment remained negative, its significant increase following fatigue suggests a shift toward greater frontal plane loading. Although the biomechanical pattern resembles a potential risk factor for ACL injury, it should be noted that this study employed a simulated landing task in healthy individuals, and no injury events were observed. Therefore, the relevance of these findings to actual injury mechanisms remains speculative. Interestingly, despite the increase in external knee abduction moment, the knee abduction angle did not significantly change. This dissociation may reflect compensatory neuromuscular control strategies that helped maintain frontal plane knee alignment in response to increased external loading. Such strategies could include enhanced muscular co-contraction or reliance on passive joint structures to resist valgus motion. These findings highlight that changes in joint moments do not necessarily accompany observable changes in joint angles, reinforcing the complexity of inferring injury risk solely from kinematic data.

**Fig. 3:**
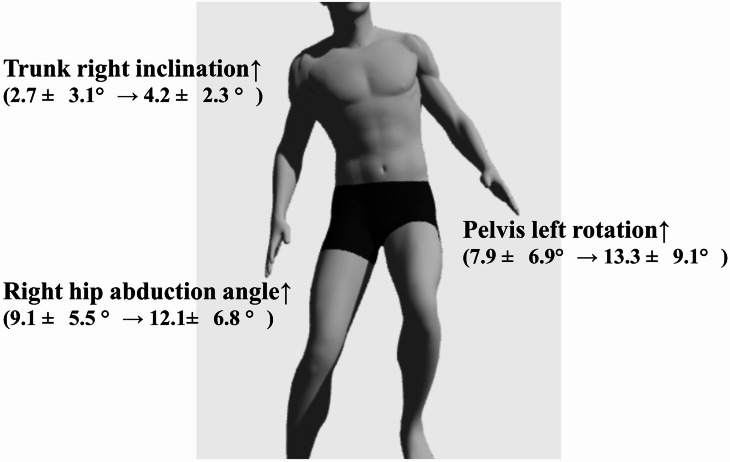
Biomechanical adaptations after targeted hip abductor fatigue during single-leg landing. Right knee abduction angle was unchanged

Traditionally, hip abductor fatigue has been conceptualized as simulating muscular weakness, potentially increasing ACL injury risk by promoting medial knee displacement^21^. However, our findings highlight a more complex phenomenon. The increase in knee abduction moment, combined with an increased hip abduction angle and compensatory trunk and pelvic adjustments, may reflect a stiffening or guarding strategy rather than simple neuromuscular insufficiency. In particular, the increase in hip abduction angle accompanied by a decrease in external hip abduction moment suggests that participants may have adopted a compensatory strategy to stabilize the pelvis and trunk without increasing active muscular force. This may reflect a shift toward passive segment positioning or altered neuromuscular coordination, potentially aiming to maintain frontal plane stability in the presence of local muscle fatigue. These adaptations could serve a stabilizing function, but may also lead to altered joint loading patterns. These multi-segmental adaptations are visually summarized in Fig. 3, which illustrates the directional changes observed in trunk, pelvis, hip, and knee biomechanics following hip abductor fatigue.

The observed increases in trunk right lean and pelvic left rotation indicate modified trunk-pelvis coordination under fatigue. These segmental deviations may help maintain balance during landing but could also introduce asymmetrical loading across the knee and hip joints. Since our analysis focused on the timing of peak vertical ground reaction force—corresponding to the early phase of landing where ACL injury typically occurs—these adaptations are particularly relevant.

Several limitations should be acknowledged. First, no direct measurement of hip abductor strength was performed, limiting our ability to quantify the degree of fatigue. In addition, baseline lower-extremity strength was not assessed, which may have influenced individual fatigue responses and adaptations. Second, the study population consisted exclusively of healthy young males, which may reduce generalizability to female athletes or clinical populations. Third, only a single post-fatigue trial was analyzed per participant, which may not capture variability in motor responses. Fourth, we did not analyze the trajectory of the center of mass (COM), which could have provided additional insights into global movement strategies during landing. Although we observed a significant reduction in the anteroposterior ground reaction force following fatigue, future studies should include COM analysis to more comprehensively evaluate fatigue-related adaptations. Finally, we performed multiple paired t-tests across several outcome variables without applying formal corrections for multiple comparisons. This approach was chosen to avoid inflating type II error in this exploratory study; however, it may increase the risk of type I error. Therefore, the results should be interpreted with caution. Despite these limitations, the present study is useful when considering the effects of hip abductor fatigue on whole-body biomechanics.

In conclusion, targeted hip abductor fatigue alters trunk, pelvic, and lower limb biomechanics during landing. While some compensatory strategies may stabilize posture, the observed increase in knee abduction moment suggests that frontal plane loading can be affected by fatigue. These results emphasize the need to interpret fatigue-related biomechanical changes in a nuanced manner and support the inclusion of hip and trunk neuromuscular training in injury prevention programs.

## Data Availability

The datasets generated and analyzed during the current study are not publicly available due to participant privacy considerations but are available from the corresponding author upon reasonable request.
